# Supplementary material for: Tectomer-Mediated Optical Nanosensors for Tyramine Determination
Source: Sensors (Basel). 2023 Feb 24;23(5):2524. doi: 10.3390/s23052524 (PMC10007293; doi:10.3390/s23052524)
Supplement: Supplementary file 1 [file sensors-23-02524-s001.zip › sensors-2191386-supplementary.pdf]

Supplementary Materials

Tectomer-Mediated Optical Nanosensors for Tyramine Determination

Mario Domínguez [1], Sofía Oliver [1], Rosa Garriga [2], Edgar Muñoz [3], Vicente L. Cebolla [3], Susana de Marcos [1,*] and Javier Galbán [1]

[1] Nanosensors and Bioanalytical Systems N&SB), Analytical Chemistry Department, Faculty of Sciences, Instituto de Nanociencia y Materiales de Aragón (INMA University of Zaragoza-CSIC), 50009 Zaragoza, Spain

[2] Departamento de Química-Física, University of Zaragoza, 50009 Zaragoza, Spain

[3] Instituto de Carboquímica ICB-CSIC, 50018 Zaragoza, Spain

S1. Quantification of Au(III) immobilized in tectomer by means of $AuBr_4^-$ complex formation.

It is known that in the presence of bromide ions, Au (III) forms a yellow-orange complex ($AuBr_4^-$) that presents a maximum absorbance at 380 nm (Figure S1a) [27]. The effect of the pH on $AuBr_4^-$ complex formation was studied, and we obtained no significant differences (P = 0.05, n = 6) from pH 2.0 to pH 6.0 (Table S1).

Table S1. Effect of the pH on $AuBr_4^-$ complex formation.

	pH 2.0	pH 5.0	pH 6.0	pH 7.0
Sensitivity ($M^{-1}cm^{-1}$)	$4.36(\pm0.05)\cdot10^3$	$4.40(\pm0.10)\cdot10^3$	$4.29(\pm0.18)\cdot10^3$	$3.80(\pm0.08)\cdot10^3$
Linear range (M)	$1.5\cdot10^{-6}$–$5.0\cdot10^{-4}$	$1.5\cdot10^{-6}$–$5.0\cdot10^{-4}$	$1.5\cdot10^{-6}$–$5.0\cdot10^{-4}$	$2.5\cdot10^{-6}$–$6.0\cdot10^{-4}$

Next, the effect of the tectomer in the formation of the $AuBr_4^-$ complex was studied. When the tectomer was added to a solution containing the $AuBr_4^-$ complex, no effect in the absorbance at 380 nm was observed. However, the addition of Br^- to a solution containing the Au(III)/tectomer complex (either at pH 6.0 or pH 7.0) led to a continuous decrease in the absorbance with the wavelength (Figure S1b) due to light scattering from the presence of tectomer aggregates in solution. In fact, the measured absorbance values were proportional to λ^{-4}, in accordance with Rayleigh-type scattering. Only a negligible (at pH 6.0) or very small (at pH 7.0)

superimposed absorbance at 380 nm was observed. In conclusion, the Au(III)/tectomer complex in solution prevents $AuBr_4^-$ formation, suggesting that strong interactions are established between Au(III) and Gly moieties in the tectomer [35, 36].

Furthermore, tectomers are known to disassemble at acidic pH values <3.0 due to electrostatic repulsion between highly protonated terminal amino groups [17]. Therefore, the Au(III)/tectomer layers prepared on PLA supports were placed inside a well-plate and 300 µL of KBr in pH 2.0 buffer solution was added to break up the Au(III)/tectomer complex. After 180 min, the resulting supernatant was characterized by measuring the UV–Vis spectra. Under these conditions, no scattering was observed and the absorbance at 380 nm was partially recovered. The solutions were also analyzed by ICP-OES, which detects both Au(III) in Au(III)/tectomer complex and Au(III) released in solution. Three samples were prepared at a 1:1 molar ratio of the Au(III)/tectomer at different concentrations, and 0.1 M KBr in pH 2.0 buffer for all samples. By comparing the results from the absorbance measurements at 380 nm and ICP-OES (Figure S1c and Table S2), it can be concluded that only 49% of the Au(III) was released from the tectomer into the solution.

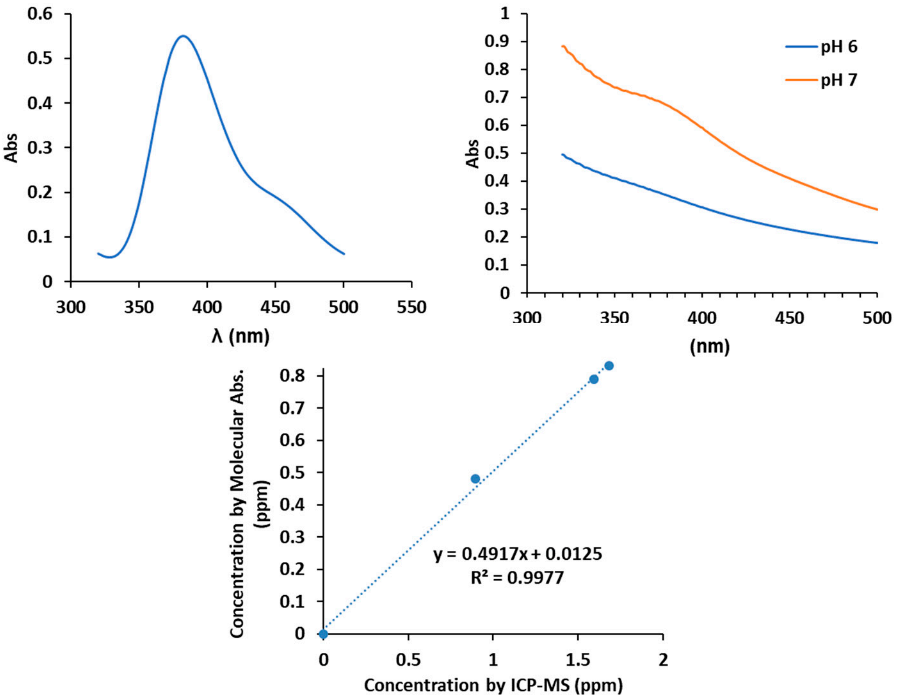

Figure S1. (**a**) Absorption spectra of $AuBr_4^-$ showing a maximum at 380 nm; (**b**) Absorption spectra upon addition of KBr to a solution containing Au(III)/tectomer complex at pH 6.0 and 7.0; (**c**) Plot representing Au(III) concentration in the supernatant resulting from the addition of KBr in pH 2.0 buffer to Au(III)/tectomer layers on PLA supports, determined from the $AuBr_4^-$ absorbance measurements at 380 nm versus the Au(III) concentration determined by ICP-MS (the slope was 0.49).

Table S2. Au(III) concentration (ppm) in the supernatant resulting from the addition of KBr in pH 2.0 buffer to the Au(III)/tectomer layers on PLA supports, determined from the absorbance measurements at 380 nm and by ICP-OES.

Sample	Absorbance	s(Abs)	ICP-OES	s(ICP-OES)
1	0.835	0.054	1.683	0.103
2	0.792	0.013	1.590	0.023
3	0.479	0.003	0.895	0.037

Interestingly, it was observed that the aging of the Au(III)/tectomer layers fabricated on PLA supports prior to the addition of KBr in pH 2.0 buffer led to a decrease in the release of Au(III) from the tectomer into the solution (Table S3). Thus, the lowest Au(III) release was obtained for Au(III)/tectomer layers aged for 6 days.

Table S3. Percentage of Au(III) released from the tectomer into the solution at pH 2.0.

Day	% Au(III) released
1	52.35
2	48.14
3	46.57
6	22.69

S2. Optimization of experimental parameters for tyramine detection using Au(III)/tectomer sensor layers.

a) Effect of the buffer solutions used in the Au(III)/tectomer premix

- pH 6.0, 7.0, 8.0, and 12.0 phosphate buffers: Tectomer aggregates formed and came out of the solution when performing the premix, which could be dissolved by bath sonication. In addition, phosphate ions favor the formation of AuNPs [37] as they increase the reduction potential of the metal.
- pH 5.0 acetate buffer: No precipitates were observed.
- pH 10.0 carbonate buffer: Faint precipitates appeared, which could be dissolved by bath sonication.
- MES buffer: A reaction takes place between the MES and Au(III) before the addition of the tectomer, which results in a black precipitate due to the reaction between the gold and sulfur, so the use of MES buffer was discarded here.

Five Au(III)/tectomer layers on PLA supports were fabricated for each pH value tested. The results obtained for the coordinate R corresponding to different concentrations of tyramine are summarized in Figure S2.

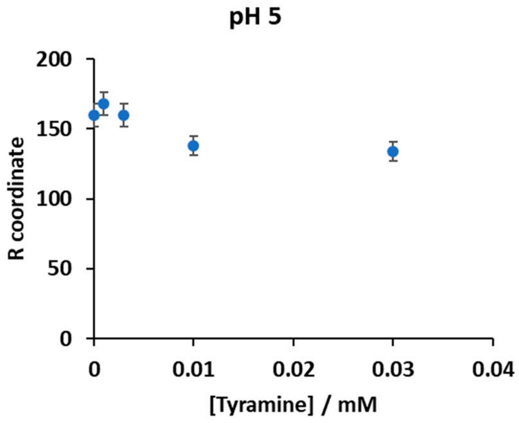

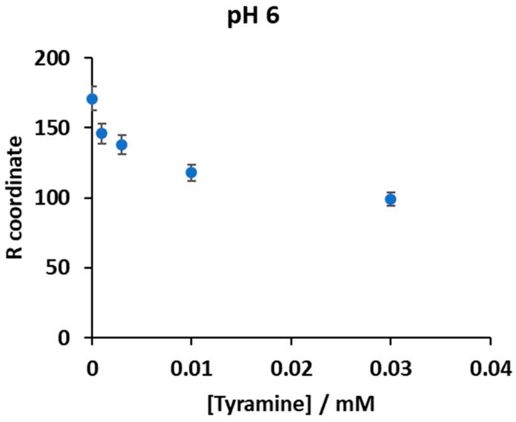

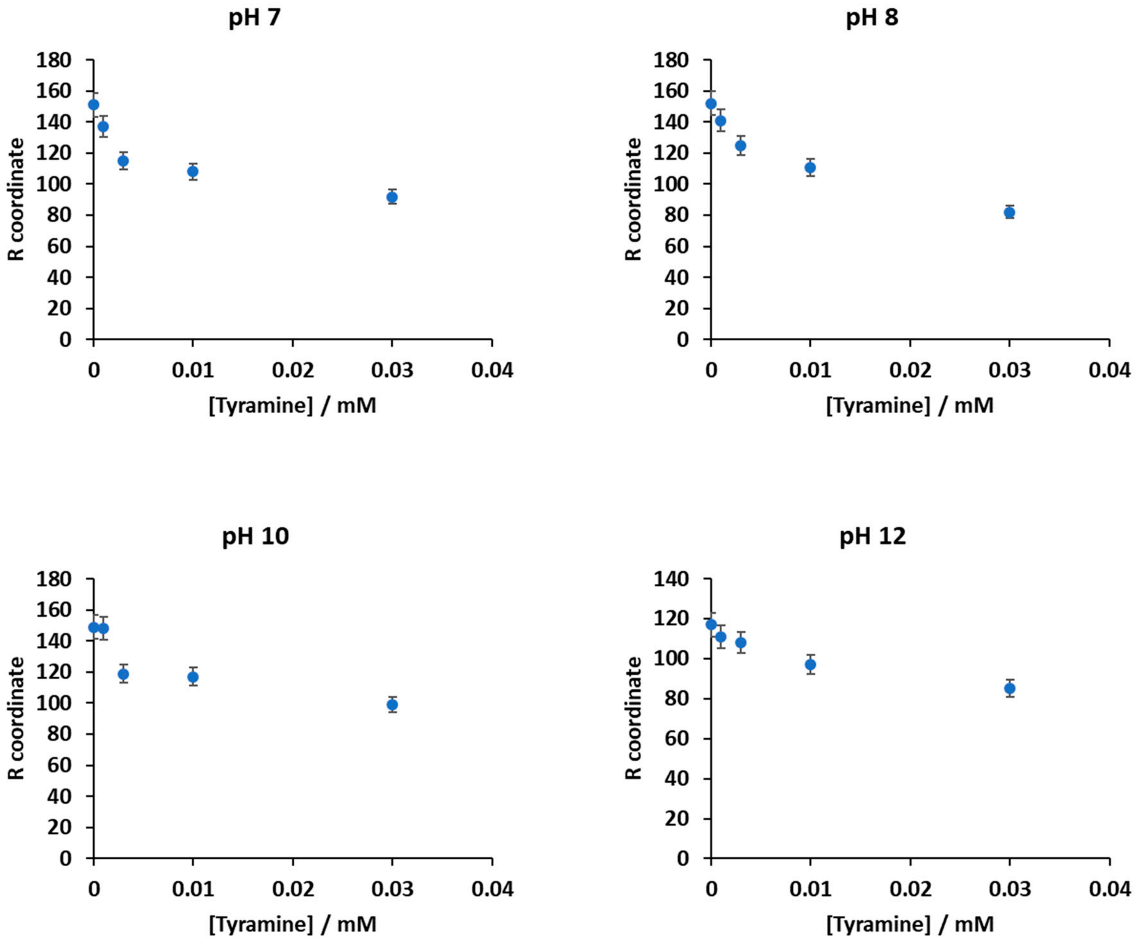

Figure S2. R coordinate as a function of the tyramine concentration for Au(III)/tectomer layers prepared using different pH buffers.

From these results, it can be concluded that the highest sensitivity was achieved using the pH 6.0 buffer.

b) Effect of the Au (III)/tectomer molar ratio

Different Au (III)/tectomer molar ratios were tested in order to obtain the best sensor response for tyramine detection (Figure S3). Five Au(III)/tectomer layers on PLA supports were fabricated for each tested molar ratio value. From these results, it can be concluded that only for the 1:1 molar ratio, the coordinate R exhibited a linear dependence on the tyramine concentration in the range below 10 μM.

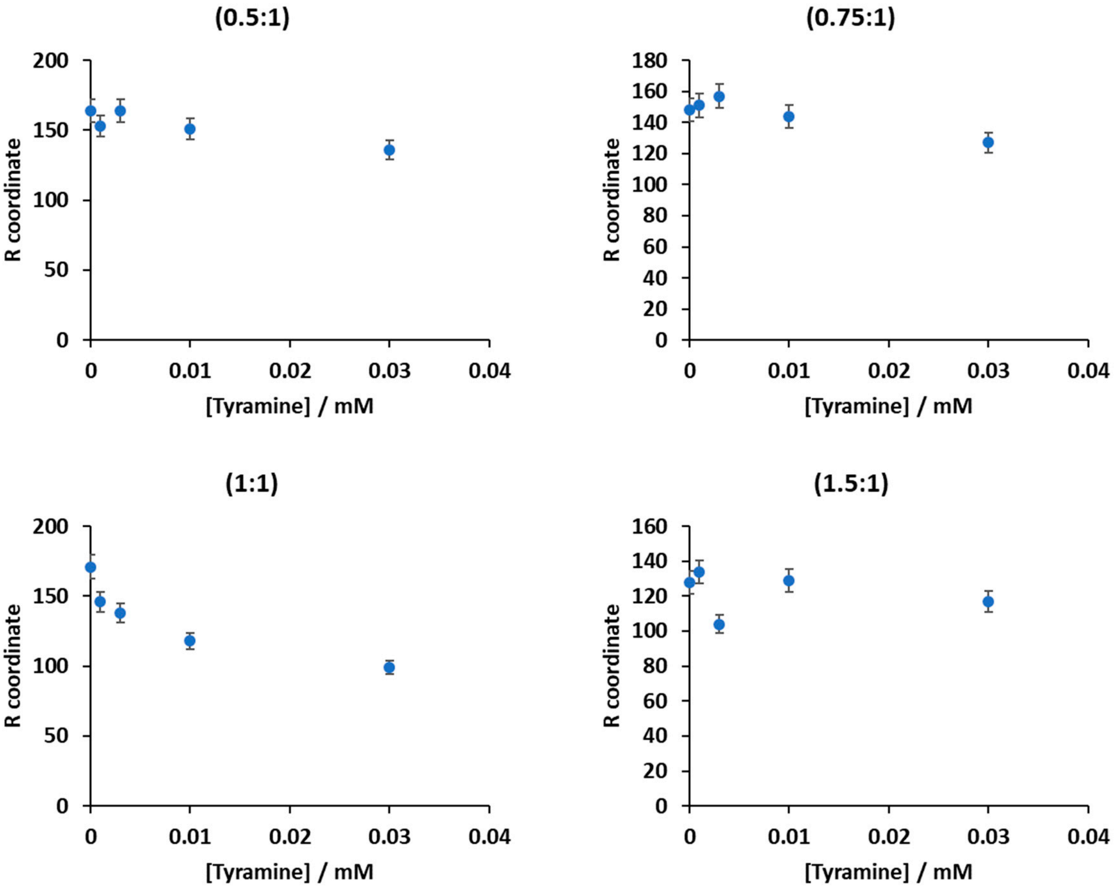

Figure S3. R coordinate as a function of tyramine concentration for different Au(III)/tectomer molar ratios.

c) Effect of the buffer solutions used to dissolve tyramine

The effect of the pH of the buffer solutions used to dissolve tyramine was studied here, as this is the pH at which the AuNPs will be formed. Figure S4 shows the Au(III)/tectomer sensing layer response when exposed to different tyramine solutions, up to 100 µM, buffered at different pH values.

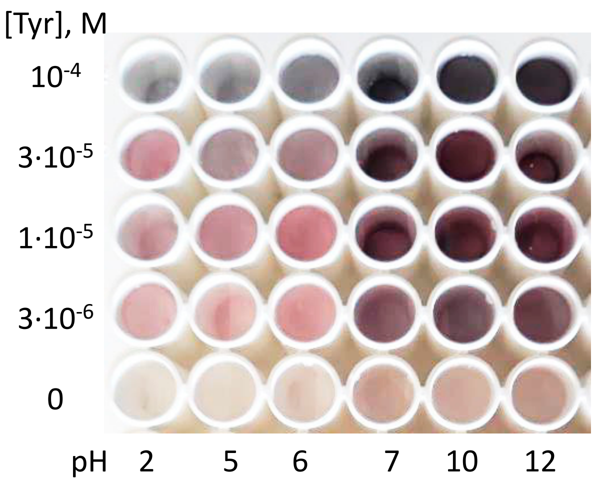

Figure S4. Au(III)/tectomer sensing layer response to several tyramine concentrations at different pH values.

Figure S5 shows that, for the 10 μM tyramine concentration, the lowest R value, which corresponded to the largest coloration change, was achieved for the pH 6.0–7.0 buffer solutions. Therefore, the tyramine detection experiments shown in the main manuscript were performed in pH 6.0 buffer.

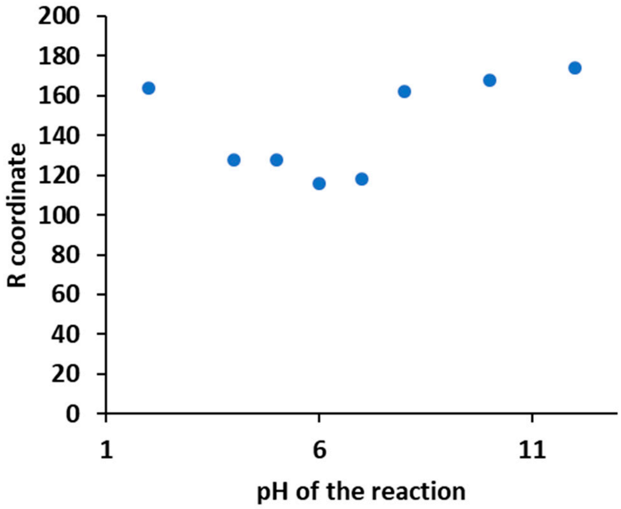

Figure S5. R coordinate as a function of the pH of the buffers used to dissolve tyramine. [Tyramine] = 10 μM in all cases.

S3. Extraction method for the cheese samples

Ten grams of cheese was weighed, crushed with 30 mL of 5% trichloroacetic acid, left to soak for half an hour, and then placed in a freezer for another half an hour. The low temperature caused the fats to solidify. The mixture was then centrifuged at room temperature for 20 minutes, at a speed of 6000 rpm. The solid phase was discarded and the supernatant solution was neutralized

by the addition of 2 M NaOH; when observing the appearance of some turbidity, a second centrifugation was carried out under the same conditions as the first. On this occasion, the supernatant was filtered through nylon membrane filters with a 0.45 μm pore diameter (ALBET-NY-045-25-BL) and collected in a 50 mL volumetric flask, whose volume was finally flushed with the pH 6 phosphate buffer. This solution was introduced into a 10K centrifuge filter (Amicon® Ultra-4) to obtain the final solution.

References

27. Kudrev, A.G. Calculation of equilibrium constants by matrix method for complexes of gold (III). *Talanta* **2008**, *75*, 380. https://doi.org/10.1016/j.talanta.2007.11.034

35. Koleva, B.B.; Zareva, S.; Kolev, T.; Spiteller, M. New Au(III), Pt(II) and Pd(II) complexes with glycyl-containing homopeptides. *J. Coord.Chem*. **2008**, *61*, 3534. https://doi.org/ 10.1080/00958970802108817

36. Glisic, B.D.; Rajkovic, S.; Zivkovic, M.D.; Djuran, M.I. A comparative study of complex formation in the reactions of gold(III) with Gly-Gly, Gly-L-Ala and Gly-L-His dipeptides. *Bioorg.Chem*. **2010**, *38*, 144. . https://doi.org/ 10.1016/j.bioorg.2010.03.002

17. Garriga, R.; Jurewicz, I.; Romero, E.; Jarne, C.; Cebolla, V. L.; Dalton, A. B.; Muñoz, E. Two-Dimensional, pH-Responsive Oligoglycine-Based Nanocarriers. *ACS Appl. Mater. Interfaces* **2016**, *8* (3), 1913–1921. https://doi.org/10.1021/acsami.5b10077.

37. Liu, K.; He, Z.; Curtin, J.F.; Byrne, H.J.; Tian, F. A novel, rapid, seedless, in situ synthesis method of shape and size controllable gold nanoparticles using phosphates. *Sci Rep* **2019**, *9*, 7421. https://doi.org/10.1038/s41598-019-43921-0
